# Exploring the experiences of residents and their families in an alcohol-related brain injury residential rehabilitation unit in Northern Ireland: a qualitative study

**DOI:** 10.3389/fpubh.2024.1397428

**Published:** 2024-11-01

**Authors:** Anne Campbell, Sharon Millen, Uisce Jordan, Diane Watson, Joy Watson, Roger McCorry

**Affiliations:** ^1^School of Social Sciences, Education & Social Work, Queen’s University, Belfast, United Kingdom; ^2^Leonard Cheshire ARBI Facility, Belfast, United Kingdom; ^3^Belfast Health & Social Care Trust, Belfast, United Kingdom

**Keywords:** alcohol related brain injury, qualitative, service user views, rehabilitation unit, substance use recovery, residential rehabilitation, Northern Ireland

## Abstract

**Objective:**

Limited research exists on comprehensive interventions for individuals with Alcohol Related Brain Injury (ARBI). Exploring the impact of a rehabilitation service on individuals with ARBI and their relatives/caregivers, this study aimed to gain insights into their experiences and assess how the service influenced cognitive functioning, psychological well-being, social relationships, community engagement, and the desire for abstinence.

**Method:**

This was a qualitative, semi structured interview study as part of a larger mixed methods study of residents and their family members. Data was collected over 4 timepoints with *n* = 20 residents: baseline (*n* = 20 interviews), 6 months (*n* = 15 interviews), 12 months (*n* = 6 interviews) and at discharge (*n* = 8 interviews). The interviews took place at a specialist residential rehabilitation facility for people with ARBI. Remote interviews were conducted with family members (*n* = 10). A thematic analysis of transcripts using NVivo software was undertaken.

**Results:**

Qualitative findings for residents with ARBI across 4 timepoints from baseline to time of discharge indicated an improvement in all outcomes. Overall, residents reported that the ARBI holistic intervention improved psychological wellbeing, social relationships/community participation, functioning abilities, and abstinence from alcohol, particularly when residents were residing in the unit. Family members and carers presented more trepidation regarding the long-term impacts.

**Conclusion:**

Whilst the residential unit provided structure and a protective environment, residents required ongoing support post discharge for their addictive behaviours. An outreach intervention for these individuals is currently being piloted.

## Introduction

Alcohol-Related Brain Injury (ARBI) is an umbrella term used to describe the damage to the structure and function of the brain caused by long term heavy alcohol consumption. ARBI symptoms are well recognized but not completely understood (1) and encompass cognitive difficulties including memory loss, trouble with performing tasks and new information processing, as well as depression, irritability, erratic behaviour, concentration issues, and impaired decision-making (2). Physical ailments like liver damage and heart issues, including hypertension and heart failure may also accompany ARBI (3). Symptoms of ARBI can range from mild to severe and many of these symptoms can improve if the person is diagnosed in time and appropriate treatment provided (4). In fact, around 75% of individuals with ARBI who receive the appropriate treatment do make some recovery with 25% making a complete recovery (5). However, since ARBI comprises a variety of conditions caused by heavy drinking, different treatment approaches have been found to help different people. It is therefore crucial that patients are assessed individually by a multidisciplinary team of professionals and a tailored approach is provided to meet the person’s specific needs (6, 7). There is a paucity of research on ARBI, and even more so research that explores the lived experience and voices of people who live with ARBI and their relatives/carers (4, 8).

The evidence base for rehabilitating ARBI is still in its infancy (9, 10). Conducting a systematic review of brain injury Bühler and Mann (11) found reversible neurodegenerative changes in heavy drinkers with sustained abstinence. To date, two types of evidence exist regarding the effect of therapeutic intervention: training in specific cognitive domains and also generic rehabilitation programmes (12). Svanberg and Evans (10) reviewed 16 studies on neurorehabilitation interventions for cognitive impairment related to ARBI, including Wernicke-Korsakoff syndrome. Most studies addressed memory impairments in Korsakoff’s syndrome. Three studies discussed service models for service development. However, the variability in methodologies and overall evidence quality limited definitive conclusions (10). Wilson et al. (13) monitored 41 patients over 25 months in a community-based phased rehabilitation programme. Among the patients, 32 achieved abstinence or were considered ‘controlled drinkers’ and placed in appropriate community settings. The study found an 85% decrease in acute hospital admissions and improvements in neuropsychiatric assessment scores. Moreover, there is some evidence regarding the efficacy of long-term care for Korsakoff’s syndrome within long term facilitates (14) and intensive inpatient neuro rehabilitation (9). Due to the scarcity of evidence on the lived experiences of patients, who often face various associated challenges such as homelessness, family dysfunction, involvement with the criminal justice system, and poor health, ARBI continues to be an inadequately researched condition. Recognizing the gaps in knowledge and clinical resources for addressing the needs of the ARBI population (15), we conducted a qualitative investigation to gather firsthand experiences of ARBI rehabilitation.

The treatment of ARBI has previously been a particularly under resourced and neglected area within Northern Ireland (NI). The Leonard Cheshire facility has developed a specialist residential rehabilitation facility for people in Northern Ireland with Alcohol Related Brain Injury. As ARBI caseloads significantly increase in acute hospitals across NI (16), the facility offers a focused and tailored approach to the post-acute treatment of ARBI patients who are referred from various pathways (see Appendix 1). This service is the first of its kind on the island of Ireland and is one element in the network of services required to support people with ARBI. Leonard Chesire was originally tasked with the development of a 14-bed residential rehabilitation facility for people in NI with ARBI for a period up to 2–3 years. The unit opened in January 2020, prior to the initial pandemic lockdown period. The intervention at Leonard Cheshire and Belfast Trust, where this research was conducted, is based upon the existing psychosocial model for rehabilitation of patients with ARBI (see Appendix 2) (13). The ARBI model specifically focuses on 5 key areas: cognitive functioning, psychological wellbeing, social relationships, community participation and maintaining abstinence through relapse prevention. The findings presented here are derived from a broader mixed methods study that examines the efficacy of an inpatient ARBI rehabilitation treatment modality in Northern Ireland. This paper specifically focuses on the qualitative findings obtained from the study. We have included qualitative interviews with residents (*n* = 20) and their carers and/or relatives (*n* = 10) to evaluate their experience of this model over a 16-month period (see Appendix 3) to gather their views on the effectiveness.

## Method

This paper specifically focuses on the qualitative findings obtained from the study which used semi structured relative/carers and service users. Ethical approval was secured from the Health and Social Care Office for Research Ethics Committees Northern Ireland (reference number: 20/NI/0108). Approval was granted in September 2020 and there was a 6-month delay in qualitative data collection due to Covid-19 pandemic and restrictions within the residential care facilities.

A purposive sampling technique was used to identify participants who were successfully referred to the ARBI facility. Twenty-three residents were invited to take part in the mixed methods research during the 16 months of data collection (data from the quantitative data collection are included in forthcoming article). Twenty residents were interviewed at baseline as three lacked capacity to participate or did not wish to take part in the interviews. The follow up interviews took place at 6 month (*n* = 15 interviews) and 12-month (*n* = 6 interviews) time points and/or at discharge (*n* = 8) (if discharged prior to these timepoints) (see Table 1). The greater proportion of residents who participated in this study were male (15/20) with an age range of 40–74 years. At the final point of data collection, residents had stayed within the unit from a period of 3 months to 30 months, with a mean stay of 15 months. Service user participants presented with a range of educational backgrounds and employment history, from unemployed to professions including, legal services and education staff (see Table 2).

**Table 1:** Qualitative interviews.

Timepoint	Number of service users
T1 baseline	20
T2 6 months	15
T3 12 months	6
Discharge	8
TOTAL	49

**Table 2:** Demographics of qualitative sample.

Participant ID	Gender	Education/ Employment	Age	Duration of stay (mths)
LC001	F	None	51	19
LC002	M	Own business	48	3
LC003	M	None	66	27
LC004	M	None	53	20
LC005	M	Worked for majority of life	70	8
LC006	F	None	64	16
LC007	M	Was in process of doing degree	52	21
LC008	M	None	51	30
LC009	M	None	48	21
LC010	M	None	54	23
LC011	M	solicitor	52	14
LC012	F	Was working at a university	51	28
LC014	M	None	65	26
LS015	F	None	51	12
LS016	M	HND	42	10
LC017	F	None	40	8
LC018	M	builder	48	10
LC019	M	None	42	5
LC020	M	None	49	5
LC022	M	Worked in armed forces	74	4

Reasons for reported escalated drinking patterns described by residents included, major surgery causing disfigurement, sudden family bereavement, domestic violence, childhood trauma and excessive social drinking.

Family members/carers (*n* = 10) of those residing in the ARBI unit were also invited to take part in an interview to capture their views and experiences regarding the service via Zoom. There were 4 males and 6 females who participated in the online interviews. Data was gathered over a 16-month timeframe between April 2021 – August 2022. All interviews, both with residents and family, were semi-structured, designed around a topic guide to ensure consistency of coverage but allowed scope for the individual circumstances. Each interview lasted between 20 and 30 min. Interviews were conducted after obtaining written consent. All interviews were audio recorded with consent, transcribed verbatim, and anonymised.

Data were analysed using the principles of thematic content analysis (17). Data analysis ran concurrently with data collection to ensure the process was as iterative as possible. An essentialist/realist approach was employed, which attempted to theorise motivation, experience and meaning in a straightforward manner. A simple largely unidirectional relationship was assumed between meaning, experience, and language (18). NVivo12 was used to assist with the organisation of data. Inter-rater checks on the semi structured interview data were carried out by two members of the research team and emerging themes and ideas were discussed and reflected upon by the team.

### Recruitment and informed consent

Participants were eligible for the study if they had been successfully referred to the Leonard Cheshire ARBI residential rehabilitation facility and had capacity to consent. Relatives/carers were eligible to participate if they were related to or had caring responsibilities for a resident within the Leonard Cheshire facility. Individuals were excluded if they did not meet any of the inclusion criteria. If a translator was available those who did not speak English could participate. All eligible participants were informed about the study by the clinical lead within the unit and were provided with accessible, written information and asked if they would consider participating. They were encouraged to read the Participant Information Sheet and there was time to ask the researcher any questions before they made their decision as to take part in the study. One eligible participant declined to participate.

This study did not recruit anyone without the capacity to consent. Patient capacity was certified by a suitably qualified clinician before data collection. The researcher also explained that the data obtained during the study would be kept secure and confidential, and that all data would be anonymised, i.e., that no one would be identifiable from the output of the study.

## Results

The ARBI model (see Appendix 3) focuses on improving cognitive functioning, psychological well-being, social relationships, community participation, and promoting abstinence. In this section, we will examine the experiences of residents and their relatives regarding these key aspects.

### Cognitive functioning

At baseline interview, almost all (19/20) residents reported varying degrees of cognitive dysfunction prior to and upon their arrival to the unit, some recalling vague memories, others remembering nothing for significant periods of time and around half experiencing some degree of confusion.

*“When I got here I just felt confused, what am I doing here? Who do I have to talk to, to get out? I know where I live but then it goes in and out as quick as it comes into my head it goes back out again … I think its starting to get better these past few weeks …” (Resident, baseline).*

At baseline, over half of residents (*n* = 12) noted that the most difficult aspect of residing in the unit was being in an unfamiliar environment away from loved ones. This impacted on the orientation and overall cognitive functioning of residents.

*“A lot of things are patchy and foggy. I am hoping if I was back in my own surroundings if you like with pictures and books, stuff that you remember from years ago that you will start remembering things or associating things …” (Resident, baseline).*

Three residents at baseline did not consider their cognitive functioning to have been affected by ARBI.

*“In terms of my cognitive functioning, I would be quite academic, I think I am quite intelligent, so I don’t think that has been impacted …” (Resident, baseline).*

#### Memory work

All residents participated in memory work and orientation skills as part of the rehab programme. At the 6-month time-point, most residents described how they had noticed improvement in their cognitive functioning; some noted improved concentration when reading, others recalled being able to remember details of TV shows, with around two thirds describing memory work to be particularly beneficial.

*“In terms of clarity I can see an improvement in terms of cognitive functioning … it has been a journey, it is still ongoing but I see the light at the end of the tunnel and I suppose I have learnt from it” (Resident, 6 months).*

Family/carer interviews revealed lingering concerns about the cognitive functioning of residents, despite acknowledging some improvement. Family members reported ongoing issues for residents who had been in the unit for a year.

*“His memory is still really bad so that is sort of still a concern that I have, his short-term memory, and I think that that is something that he has to live with” (Relative 5).*

At the 6-month and 12-month time points, residents had noticed a change in their overall functioning abilities. In addition to memory work, residents were encouraged to engage in orientation work to enable them to recognize the time of day, their location and how to journey to and from the shop. Residents also had daily activities within the unit, on a rota basis. Through completing these activities, all residents reported that there was a notable improvement in their daily living and competence in key life skills.

A key factor in rehabilitation and recovery highlighted by residents, particularly at the 12-month time point, was the benefit of establishing and maintaining a structured routine. Most residents (4/6) described how engaging in a structured routine which involved completing daily tasks and activities provided them with a sense of purpose and wellbeing. Over half of (*n* = 10) relatives/carers of the residents reported there to be a significant improvement in terms of the ability of their loved ones to carry out tasks required for independent daily living, specifically daily hygiene and looking after personal space.

*“I think he’s able to tidy his room, do his own washing, do a wee bit of vacuuming, and wash himself, I ask him what goes on when I go up on a Saturday and I don’t get much, he doesn’t really say much sometimes, he’ll say that there was a quiz on or maybe they were watching the football” (Relative 6).*

### Psychological wellbeing

Another key aim of the rehabilitation service was to promote emotional wellbeing, manage mental health conditions and build resilience and self-confidence. At baseline, around a quarter of residents found it difficult to articulate how they felt psychologically. There was a range of responses from trepidation to frustration to generally feeling low. Some residents articulated their diagnoses of anxiety and depression as comorbid conditions alongside ARBI. Almost all interviewees reported an improvement in their overall mood, particularly their emotional wellbeing at the six and twelve-month time points. All attributed this improvement to the rehabilitation programme and staff.

*“I feel as though there has been a fantastic improvement in my mood and overall health. When I first came in, God forgive me but I was like a zombie, I didn’t know where I was or what I was seeing, what I was watching, what I was listening to. They work with you and kind of school you and they have brought me around now that I can turn around and say there is a great film on Saturday night, we will all go and watch it. Whereas a few weeks ago I wouldn’t have known what was on or what the TV was” (Resident, 6 months).*

Most relatives/carers acknowledged some degree of improvement in the psychological well-being of their loved ones, attributing it to their current abstinence from alcohol, improved sleep and nutrition, structured routines, and the care provided by the staff. Relatives/caregivers identified company as a contributing factor to improved psychological well-being. However, some relatives/caregivers mentioned that their family member faced additional complex issues, such as PTSD, hoarding, and eating disorders, making it more challenging to assess a significant improvement in psychological well-being.

*“Her mental health, I don’t think she will ever be well because she is still hoarding massively, she had such a huge problem with that. I had to clean her house out, it took a week, we had to hire a skip, she had stuff from 20 years. It was just crazy, but she is doing that there (in unit). She is collecting 100s and 100s of little stones and washing them with a toothbrush and leaving them all over so her mental health is not good; her hoarding has always been there… she just got way out of hand as her mental health deteriorated” (Relative 4).*

### Social relationships and group participation

The rehabilitation service aimed to promote resident’s engagement with both internal and external social support networks and foster a sense of community within the facility. This involved encouraging residents to interact informally and participate in shared activities, such as weekly meetings, to cultivate stronger social networks, access support, and develop friendships. At baseline, one-third of interviewees described the difficulties encountered living in a communal environment. Some were apprehensive of other residents as they had been used to living alone and spending most of their time in isolation. Moving to a shared space with 13 other residents and a staff team required a high level of adjustment.

*“I am used to living on my own, so it’s hard to live with so many different people. So, coming into this is a shock, sitting in a full dining room it’s like eating out every mealtime” (Resident, baseline).*

During the initial settling-in phase, a quarter of residents expressed a preference for spending time alone in their rooms rather than socializing and getting acquainted with other residents. This was due to several factors such as fear, anxiety, confusion, and unfamiliarity of socializing with others. At the 6-month time point (*n* = 15) however, most residents had adapted and felt at ease in their shared environment, reporting increased integration and social connections within the unit.

*“When I first came in … I never bothered much, I would have went and made a cup of tea but everyone would be having a cup of tea and I would have walked past them and straight back up to the room … whereas now I’m knocking at doors to see if anyone is going for a cup … for a chat and it’s all down to the staff in here for bringing that out of me …” (Resident, 6 months).*

At the 12-month time point, all residents (*n* = 6) had developed strong bonds and close friendships within the unit, contributing to a relaxed atmosphere that facilitated their positive recovery journey. Weekly in-house resident meetings served as a formal platform for residents to connect, exchange information, and engage socially, with approximately half of the residents considering these meetings as beneficial for learning, sharing opinions, and socializing with fellow residents. The purpose of these meetings is to prepare residents for life in the community after discharge.

Regarding discharge, around one-third of residents required additional support due to comorbidities, which needed to be taken into consideration when attempting to find suitable placements for before discharge. Residents reported concerns regarding the discharge process. Over half of residents at time of discharge (5 out of 8) reported delays around securing suitable placements for those who had completed their rehabilitation programme.

#### Engagement/relationship with staff

Residents emphasised the significant role of their relationship with the staff in their ongoing recovery, with approximately two-thirds highlighting that the staff treated them without judgment, considering them as “normal.” This non-judgmental approach was regarded as a crucial factor in their recovery.

*“They treat you as a normal person in here, they don’t treat you as someone who has got a disease or got something wrong” (Resident, 6 months).*

Residents consistently highlighted the exceptional support, friendliness, and approachability of the staff across all timepoints. This rapport played a crucial role in fostering trust and enabling residents to actively participate in the programme. Some residents also praised the staff for giving them time out when needed and encouraging autonomy over their daily schedule.

*“The staff are great, they have helped me to improve and to become more independent by doing more things for myself. In the last place I was in I wasn’t doing a thing for myself” (Resident, 12 months).*

*“They don’t tell you too much in here so you will have to work it out yourself, you know … I would like to know the whole story. Where was I? Was I behaving?… I was in hospital for a wee while, I was taken out and, they don’t tell me very much … You ask things but then they don’t like to tell you too much. So you are left … I sort of want it all laid out in front of me … You are always looking for more to make things less confusing” (Resident, discharge).*

#### Engagement with family and significant others

Social interaction with family and friends was promoted in the unit. Approximately one-third of residents (*n* = 7) experienced distress and confusion regarding the status of personal relationships at baseline. Respondents described how they felt fearful about their actions because of drinking to excess prior to their arrival in the unit. Whilst some could not recall if they had damaged relationships, others were aware that they had done so and expressed remorse. One resident noted that it would be beneficial for them to have more information regarding their condition, its severity, as well as an estimated time frame of stay in the unit.

Around half of residents at 6 and 12-month time points perceived their relationships to have improved with family and friends because of their time in the unit and professional help.

*“My relationship with my daughters has changed a hell of a lot. I’m not grumpy, I’m not short tempered, I’m more patient and I can have a good decent conversation with them. From being in here that has helped me to improve my relationship … It’s a different situation now and it’s 100% better than what it was before being here, it’s getting the professional help, that’s the big part of it” (Resident, 6 months).*

Others did not perceive any significant improvement as they felt their relationship to have been strong from the outset. This was reiterated by around a third of relatives/carers who felt that their positive relationship with their relative had not changed significantly.

### Desire to remain abstinent

Another key objective of the service was to increase the ability of the residents to maintain abstinent. During the interviews, residents were asked about their perspectives on maintaining abstinence after discharge and how they perceived managing this outside of the unit. With one exception, all participants consistently expressed a strong desire to remain abstinent from alcohol upon discharge. The majority shared how their lives had significantly improved during their time in the unit and their alcohol-free period, reporting improvements in both physical and mental health. Many also expressed their motivation to stay sober, considering the burden it placed on their loved ones.

## Discussion

ARBI has a significant impact on cognitive function, making it a key area of focus within the rehabilitation unit. All residents interviewed at 12 months agreed that there had been a significant improvement in their cognitive functioning since their arrival. However, participants noted the challenge of an unfamiliar environment impacting memory and recall, aligning with Mimura et al.’s (19) study. Family members were less optimistic about cognitive improvements and expressed concerns about the lack of perceived positive improvement in cognitive function for their loved ones. Residents are expected to improve over time and require regular review and adjustment of care plans to optimise rehabilitation (13, 20).

Positive attitudes towards social relationships and engagement were highlighted in the long term with respondents reporting the development and maintenance of friendships with other residents at the 12-month juncture. Participants unanimously appreciated the respect shown towards residents in the LC facility, emphasising the absence of judgment based on their personal history, which is especially significant given the stigma associated with ARBI (4). Engaging in daily activities within the unit facilitated the reacquisition of essential skills for independent living and contributed to a restored sense of self-worth. Research findings support the effectiveness of a broader rehabilitative approach that emphasises social and behavioural aspects, on par with specific cognitive interventions, in facilitating improvement (21, 22). Residents and their relatives/carers reported positive improvements in psychological well-being, attributing them to factors such as abstinence from alcohol, regular sleep patterns, good nutrition, and a structured daily routine [*cf.* (23, 24)]. Some carers/family members noted the challenge of observing improvements in this area due to the presence of complex comorbidities.

Residents and their relatives/carers were asked for feedback on how to enhance their experiences in the unit, and the responses mainly centred around practical support, increased activities, and improved facilities. Relatives/carers and residents expressed the need for more information about ARBI. Suggestions included an informative pack for families to understand the condition and support their loved ones, as well as providing residents with details about the condition’s severity and expected duration of their stay in the unit. Relationships with staff were seen as pivotal to the success of the participants’ rehabilitation and increased social, physical, and cognitive functioning. Alongside help in achieving abstinence (8, 25, 26), the provision of family and social-based support and structure is crucial (27, 28).

The service aimed to reintegrate residents into the community with the highest level of independence achievable after completing their rehabilitation programmes. The specific outcome varied based on individual resident’s needs, ranging from returning to independent living in a bungalow to residing with family or transitioning to assisted living facilities. Blansjaar et al. (29) found patients did continue to improve, especially if placed in smaller institutions. The important clinical issue is that patients are likely to improve over time and will need regular review and adjustment to care plans to optimise rehabilitation (13, 20). People with ARBI often require additional support due to comorbidities, which must be considered when seeking appropriate post-discharge placements for residents (30). However, residents consistently reported delays in securing suitable post-rehabilitation placements, particularly when expressing a desire to live in specific areas of Northern Ireland. When asked about potential improvements, both residents and family members/carers unanimously agreed that addressing this issue should be a top priority.

Limited understanding of ARBIs and related services hinders recovery progress by contributing to increased challenging behaviour and reduced engagement (13, 31). We recommend a focus on increasing the availability of assisted living placements and the development of a Step-Down facility to facilitate the transition from discharge into the community. Given that most patients with ARBI show some recovery with treatment, a process of screening and diagnosis is required to actively identify them and engage them with clinical and support services (7, 32, 33). Expanding the current outreach service to include community referrals for individuals not yet requiring hospitalisation would aim to decrease the need for inpatient treatment and reduce costs for the NHS.

There is evidence for specialised and person-centred treatment for people with ARBI, focusing on cognitive remediation or rehabilitation (4, 13, 21, 34) and this study aims to inspire future developments in this area. People with ARBI have great potential for recovery and leading fulfilling lives. Researchers and clinicians have a responsibility to develop treatment programmes and services to support this potential. The evidence which highlights the success of the unit should be used to support the establishment of further units in Northern Ireland. Further research should focus on estimating the nature and extent of ARBI in NI.

## Data availability statement

The raw data supporting the conclusions of this article will be made available by the authors, without undue reservation.

## Ethics statement

The studies involving humans were approved by Health and Social Care Office for Research Ethics Committees Northern Ireland (reference number: 20/NI/0108). The studies were conducted in accordance with the local legislation and institutional requirements. The participants provided their written informed consent to participate in this study.

## Author contributions

AC: Conceptualization, Funding acquisition, Supervision, Writing – original draft. SM: Formal analysis, Investigation, Project administration, Writing – original draft. UJ: Investigation, Methodology, Validation, Writing – review & editing. DW: Data curation, Resources, Visualization, Writing – review & editing. JW: Project Administration, Investigation, Writing – review & editing. RM: Funding acquisition, Supervision, Writing – review & editing.
