# Supplementary material for: Exploring the experiences of residents and their families in an alcohol-related brain injury residential rehabilitation unit in Northern Ireland: a qualitative study
Source: Front Public Health. 2024 Nov 1;12:1397428. doi: 10.3389/fpubh.2024.1397428 (PMC11563969; doi:10.3389/fpubh.2024.1397428)
Supplement: Supplementary file 1 [file Data_Sheet_1.docx]

### Appendix 1: Care Pathway for People with ARBI

Care Facilities

Community Services

Hospital

Trust Care Management

Addictions services/community addictions team

**Leonard Cheshire ARBI Unit**

Outreach Service

Independent living with access to community-based services

Community living with support services e.g., floating support

Supported Living
