# Supplementary material for: Exploring the experiences of residents and their families in an alcohol-related brain injury residential rehabilitation unit in Northern Ireland: a qualitative study
Source: Front Public Health. 2024 Nov 1;12:1397428. doi: 10.3389/fpubh.2024.1397428 (PMC11563969; doi:10.3389/fpubh.2024.1397428)
Supplement: Supplementary file 2 [file Data_Sheet_2.docx]

### Appendix 2: Therapeutic phases of rehabilitation in ARBI

| Therapeutic phases of rehabilitation in ARBI.  ***Wilson. et. al. (2012) The Psycho-Social Rehabilitation of Patients with Alcohol Related Brain Injury (ARBI) in the Community*** | | |
| --- | --- | --- |
| **Phase** | **Characterisation** | **Duration** |
| **1.Stabilisation** | *Acute withdrawal, management of encephalopathy, thiamine supplementation, physical stabilisation.*  *Usually carried out inn acute medical wards* | *Variable; dependent upon physical health* |
| **2.Psycho-Social Assessment** | *Evidence of fairly rapid improvement in cognitive and behavioural profile. Period of ongoing assessment in a therapeutic environment. Introduction of early routine, structure, and support.*  *Regularisation of sleep, appropriate nutritional maintenance, and mood stabilisation.*  *Development of therapeutic relationships.*  *Early engagement with family and carers* | *May last up to three months. Duration may be increased when complicated by other organic or psychiatric conditions* |
| **3.Therapeutic Rehabilitation** | *Period of more gradual improvement in cognitive and behavioural skills.*  *Milieu based approach.*  *Rehabilitation in an ecologically relevant, adaptable environment.*  *Focus on cognitive, emotional, social behavioural and functional skills development.*  *Development of social relationships and therapeutic relationships.* | *May last up to three years. Ca be complicated by co-morbid physical and mental illnesses.* |
| **4.Adaptive Rehabilitation** | *Rate of cognitive and behavioural improvement has slowed or ceased; social and physical environment is adapted to optimise independence by compensating for residual cognitive and functional deficits* | *Duration may vary dependent on personal circumstances and access to facilities* |
| **5.Social Integration and Relapse Prevention** | *Building new social relationships, structured routines, and alcohol relapse prevention* | *Long term follow-up required.* |
