# Supplementary material for: Exploring the experiences of residents and their families in an alcohol-related brain injury residential rehabilitation unit in Northern Ireland: a qualitative study
Source: Front Public Health. 2024 Nov 1;12:1397428. doi: 10.3389/fpubh.2024.1397428 (PMC11563969; doi:10.3389/fpubh.2024.1397428)
Supplement: Supplementary file 3 [file Data_Sheet_3.docx]

### Appendix 3: Model for ARBI Unit
